# Long Term Immune Responses to Pandemic Influenza A/H1N1 Infection in Solid Organ Transplant Recipients

**DOI:** 10.1371/journal.pone.0028627

**Published:** 2011-12-14

**Authors:** Aliyah Baluch, Atul Humar, Adrian Egli, Jonathan Gubbay, Luiz Lisboa, Leticia Wilson, Deepali Kumar

**Affiliations:** 1 Alberta Institute of Transplant Sciences and Li Ka Shing Institute of Virology, University of Alberta, Edmonton, Alberta, Canada; 2 Ontario Agency for Health Protection and Promotion, Toronto, Ontario, Canada; 3 University of Toronto, Toronto, Ontario, Canada; University of California Los Angeles, United States of America

## Abstract

In solid organ transplant (SOT) recipients it is unknown if natural infection with influenza confers protection from re-infection with the same strain during the next influenza season. The purpose of this study was to determine if infection with pandemic influenza A/H1N1 (pH1N1) resulted in a long-term immunologic response. Transplant recipients with microbiologically proven pH1N1 infection in 2009/2010 underwent humoral and cell-mediated immunity (CMI) testing for pH1N1 just prior to the next influenza season. Concurrent testing for A/Brisbane/59/2007 was done to rule-out cross-reacting antibody. We enrolled 22 adult transplant patients after pH1N1 infection. Follow up testing was done at a median of 7.4 months (range 5.8–15.4) after infection. After excluding those with cross-reactive antibody, 7/19 (36.8%) patients were seroprotected. Detectable pH1N1-specific CD4+ and CD8+ interferon-γ producing T-cells were found in 11/22 (50%) and 8/22 (36.4%) patients respectively. Humoral immunity had a significant correlation with a CD4 response. This is the first study in transplant patients to evaluate long-term humoral and cellular response after natural influenza infection. We show that a substantial proportion of SOT recipients with previous pH1N1 infection lack long-term humoral and cellular immune responses to pH1N1. These patients most likely are at risk for re-infection.

## Introduction

Pandemic influenza A/H1N1 (pH1N1) caused widespread infection in 2009 and early 2010 creating a spectrum of disease in organ transplant recipients with a mortality rate of up to 7.8% [1]–[3]. An important clinical question in transplant patients who were infected with pH1N1 during the initial pandemic was whether they would be at risk for re-infection with pH1N1 in the subsequent influenza season. Humoral and cellular responses to influenza infection are likely important in determining disease severity and recovery from infection. The humoral response to influenza includes the development of neutralizing antibodies against the surface glycoprotein, hemagglutinin. This antibody response is seen at 4 to 7 weeks post-infection and declines slowly afterwards. One study showed a 100% seroconversion rate to pH1N1 infection in healthy 14 to 20 year olds by day 30 post-infection. Antibody titers were present in only 52% of patients by day 180 [4]. Although the antibody response is very important in subsequent protection against infection, CD4+ and CD8+ T-cell responses also play a role [5], [6]. Cytotoxic T lymphocyte (CTL) response to influenza has been shown to peak at 14 days post infection in immunocompetent individuals [5]. A CTL response is directed towards the internal conserved proteins of the virus and reduces the severity of disease although has not been shown to prevent disease. CD8+ T cell response has correlated with reductions in the duration and level of virus replication in adults who have a history of low levels of antibodies that are then challenged with seasonal influenza A. However, this cellular immunity has been shown to diminish over years [7]. Critically ill patients with pH1N1 have also demonstrated strong interferonγ, T-helper (Th) 1 and Th17 response to infection early in the course of illness although the long-term sustainability of these responses is not known [8].

It is also unknown if organ transplant recipients are able to produce similar humoral and cellular responses to pH1N1 infection compared to immunocompetent persons. Equally, it is unknown whether transplant recipients that recover from influenza infection retain a long-term humoral response or have a robust cellular response if rechallenged with the same viral subtype. Seasonal influenza vaccine responses in transplant recipients are known to be suboptimal. Monovalent pandemic vaccine responses in transplant recipients have been shown to be similarly low [9]. Therefore, similar to vaccination, we hypothesized that transplant recipients would have poor long-term immunity to natural influenza infection and would therefore be at risk of being re-infected with the same strain during the next influenza season. The purpose of our study was to determine whether organ transplant recipients retain specific immunity to pH1N1 several months after infection.

## Methods

### Patient population

This study was approved by the institutional Ethics Review Board. All patients provided informed consent. Adult organ transplant recipients seen at the University of Alberta Hospital, Edmonton, were prospectively enrolled in the study if they had microbiologically proven pH1N1 during 2009–2010. All influenza A positive specimens were confirmed as pH1N1 by PCR. Serum and peripheral blood mononuclear cells (PBMCs) were collected from each patient prior to the onset of the next influenza season (2010–2011 season). Clinical information collected included demographic data, hospitalization due to the original pH1N1 infection, treatment of infection, and type of immunosuppression.

### Laboratory Methods

Serum and PBMCs were collected from transplant recipients with previous pH1N1 infection. Sera were stored at −80°C and underwent a hemagglutination inhibition assay (HAI) at the Ontario Agency for Health Protection and Promotion, Toronto, Ontario using a previously described method [10]. Sera underwent HAI for A/California/7/2009 and for A/Brisbane/59/07 to rule out cross-reactive H1N1 antibody. The HAI assay was performed with 0.7% guinea pig erythrocytes and 4 HA units of virus. Sera were tested at an initial dilution of 1∶10 and a final dilution of 1∶1280. PBMCs were isolated from whole blood using Ficoll-Paque Premium (Pharmacia, Uppsala, Sweden) gradient density centrifugation and stored in liquid nitrogen till use. To measure intracellular IFNγ responses from pH1N1-specific T-cells, PBMCs were thawed, then incubated for 16 hours at 37°C in 5% CO_2,_ counted and adjusted to a concentration of 1×10^6^ cells per 400 µL. The samples were then stimulated for 24 hours with live pH1N1 virus (A/California/4/2009; Advanced Biotechnologies Inc., Columbia, MD) using a multiplicity of infection (MOI) of 0.5 and 1×10^6^ cells per reaction. Anti-CD3 (10 µg/mL) was used as the positive control; media alone served as the negative control. Brefeldin A was added 2 hours after stimulation to halt further interferonγ (IFNγ) secretion. Using fluorescent monoclonal antibodies (eBioscience Inc., San Diego, CA), the cells were surface stained for CD4 and CD8 with anti-human CD4 (PE-Cy7) and anti-human CD8 (APC-eFluor 780) respectively. After fixation, PBMCs were stained for intracellular interferonγ with PE. The percentage of pH1N1-specific CD4+ and CD8+ T cells producing interferonγ was determined using flow cytometry [11]. The flow data were acquired using a FACSCanto instrument equipped with FACSDIVA software and the results were analyzed by FCS Express Version 3 (from De Novo Software). 50,000 lymphocyte gate events were obtained per single sample. Isotype control antibodies and negative control samples were used to define the amount of non responsive T-cells. Prior to testing patient samples, the protocol was validated using a) PBMCs from non-transplanted persons who were vaccinated or had microbiologically-confirmed pH1N1 during 2009–2010 (positive controls for the method); and b) PBMCs from healthy volunteers stored during 2008 prior to the onset of pH1N1. These served as negative controls for the CMI protocol. In 16 healthy individuals either vaccinated or with previous pH1N1, the median CD4+ T-cell frequency was 0.92% (range 0.35–3.2) and the median CD8+ T cell frequency was 0.96% (range 0.33–2.43). This compared to three negative controls with median CD4+ and CD8+ T cell frequency of 0.03% and 0.04% respectively.

### Definitions in the study

For the humoral response post-infection, seroprotection was defined as a strain-specific antibody titer ≥1∶40 [12]. A/Brisbane/59/2007 (also an H1N1 virus) is a seasonal strain used in the annual trivalent influenza vaccine for 2009–2010. Since influenza A/Brisbane may result in cross-reactive antibody to pH1N1, if sera also showed a titer ≥1∶40 to influenza A/Brisbane, seroprotection was only considered if pH1N1 antibody titer was 4-fold greater than A/Brisbane titer (even though patients may also have seroprotective levels to pH1N1). For the pH1N1-specific cellular immune response post-infection, CD4+ IFNγ production was calculated by dividing the number of CD4+ cells producing IFNγ by the total number of CD4+ T-cells. The same calculation was done for CD8+ IFNγ production. A positive pH1N1 specific CD4 or CD8 IFNγ response was defined as ≥2 standard deviations above the mean of the negative control for each sample and greater than 0.2%.

### Statistics

Statistical analysis was performed using SPSS (version 18.0; SPSS) and GraphPad Prism version 4.0. A univariate analysis was performed to determine variables affecting humoral and cellular responses. Correlations between humoral and cellular immunity were assessed using linear regression.

## Results

### Patient population

During 2009–2010, 25 adult solid organ transplant recipients had microbiologically proven pH1N1 at our center. Three patients were lost to follow-up or did not provide consent for blood testing. Therefore, we were able to enroll 22 adult patients for follow-up blood prior to the 2010–2011 influenza season. Baseline characteristics of the cohort are shown in Table 1. The four most common types of transplants were lung (7/22; 31.8%), kidney (6/22; 27.3%), heart (3/22; 13.6%), and liver (3/22; 13.6%). The median time from transplantation to documented pH1N1 infection was 4.5 years (range 0.3 to 18.7). The most common combination of immunosuppression used included mycophenolate mofetil (MMF), calcineurin-inhibitor, and prednisone. Antiviral therapy (oseltamivir) was given to 21/22 (95.5%) patients and 7/22 (31.8%) patients required hospitalization. Only 2/22 (9.1%) recipients required ICU admission while hospitalized. Six patients had chest x-ray findings consistent with pneumonia during their hospitalization. Following recovery from pH1N1 infection, patients were told by their transplant teams they should not receive the monovalent pH1N1 vaccine as they had already had resolved natural infection. However, three patients received the monovalent pH1N1 vaccine during 2009/2010 influenza season and of these, two were immunized prior to infection and only one patient was immunized within one week of resolved infection. For seasonal vaccination, patients were encouraged to receive the 2009/2010 seasonal trivalent vaccine. At the time of testing, no patient had yet received annual influenza vaccine for the 2010–2011 season.

**Table 1:** Baseline characteristics of cohort with microbiologically proven pandemic influenza A/H1N1 during 2009/2010.

Characteristic	N = 22 (%)
Gender (M/F)	14/8
Median age (years)	55.5 (range 26–71)
Type of Transplant	
Lung	7 (31.8%)
Kidney	6 (27.3%)
Heart	3 (13.6%)
Liver	3 (13.6%)
Other (Combination or Islet)	3 (13.6%)
Time from Transplant to Infection (median and range)	4.5 years (range 0.3–18.7)
Time from Infection to Follow-up Testing (median and range)	7.4 months (range 5.8–15.4)
Immunosuppression	
MMF	19 (86.4%)
Calcineurin-inhibitor	19 (86.4%)
Prednisone	16 (72.7%)
Sirolimus	1 (4.5%)
Azathioprine	1 (4.5%)
Everolimus	1 (4.5%)

### Humoral Response

The median time from natural infection to assessment of humoral immune resonse was 7.4 months (range 5.8 to 15.4). Of the cohort, 10/22 (45.5%) patients met the criteria for long-term seroprotection to pH1N1 (≥1∶40 antibody titer). In 5/22 cases, patients also had cross-reactive antibody to A/Brisbane. In three cases, pH1N1 antibody titers did not exceed A/Brisbane titers by ≥4-fold; therefore, these results were considered indeterminate and patients were excluded from further analysis. Therefore, 7/19 (36.8%) patients were confirmed seroprotected for pH1N1. In these 7 patients, the median titer was 1∶160 (range 1∶40 to 1∶640). Patients hospitalized for pH1N1 were more likely to have a long-term seroresponse 4/5 (80%) than those not admitted to hospital 3/14 (21.4%); p = 0.038. Thoracic (heart and lung) transplant recipients had a trend towards a lack of long-term humoral response to pH1N1 infection compared to other SOT recipients (11.1% vs. 60.0%; p = 0.057). Shorter time to follow up of serologic measurement after infection was associated with a higher likelihood of seroprotection (7.5±2.2 months in responders vs. 9.1±2.6 months in non-responders, p = 0.047). For patients who were measured between 5–8 months post-infection, the seropositivity rate was 50% (6/12); for those measured between 8–12 months (seropositivity was 0% (0/5)) and for those between 13–15 months (seropositivity was 50% (1/2)). Factors that were not associated with a sustained humoral response included time from transplant, type of immunosuppression or calcineurin-inhibitor levels, early versus late antiviral treatment, presence or absence of lymphopenia and history of previous seasonal influenza vaccination. Of the three patients that were also immunized for pH1N1, only one had sustained seroprotection.

### Cell Mediated Immunity (CMI)

**Figure 1:**
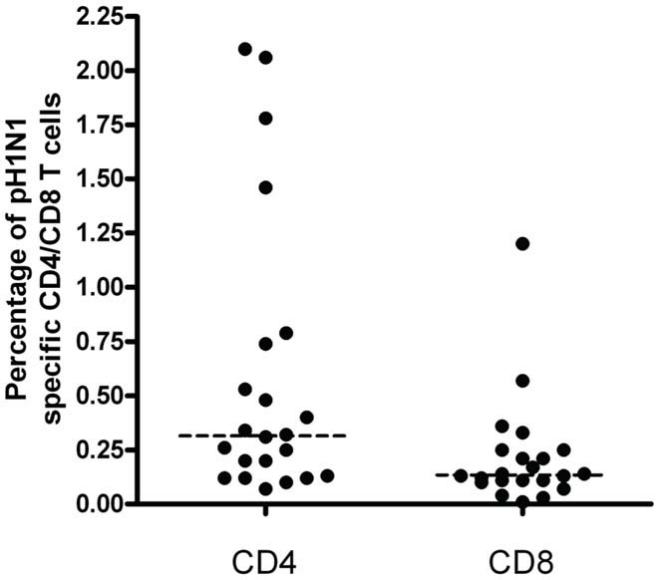
Range of pH1N1-specific CD4+IFNγ+ and CD8+IFNγ+ T cell frequencies (%) in peripheral blood mononuclear cells of individual patients (n = 22). Samples taken after natural pH1N1 infection but prior to the onset of the next influenza season. Horizontal line represents median response.

**Figure 2:**
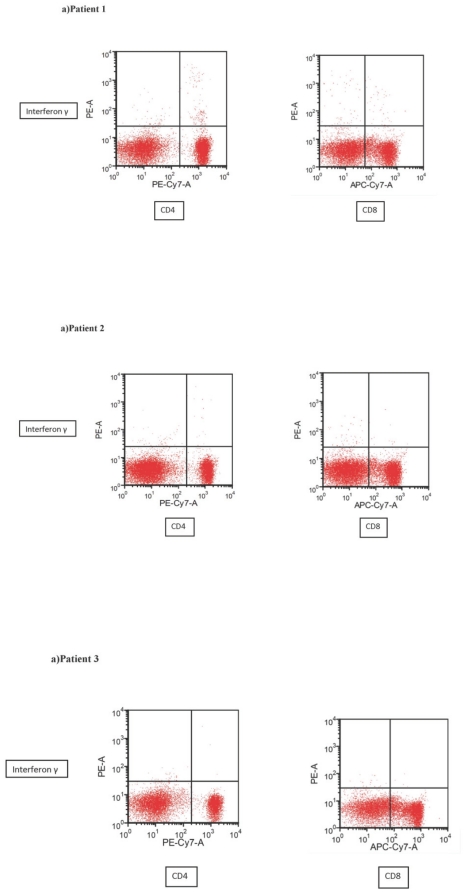
Representative flow cytometry plots for 3 individual patients. A) Patient 1: pH1N1 antibody titer of 1∶640, CD4+/IFNγ frequency of 1.78% (positive), and CD8+/IFNγ frequency of 0.57% (positive); B) Patient 2: pH1N1 antibody titer of 1∶80, CD4+/IFNγ frequency of 0.25% (negative), and CD8+/IFNγ frequency of 0.13% (negative); C) Patient 3: Negative serology, CD4+/IFNγ frequency of 0.07% (negative) and CD8+/IFNγ frequency of 0.07% (negative).

Similar to the antibody assessment, the median time from infection to assessment of CMI was 7.4 months. All 22 patient PBMCs underwent flow cytometry analysis for pH1N1-specific IFNγ production from CD4+ and CD8+ T cells. The range of pH1N1 specific T cell frequencies is shown in Figure 1. The median CD4+IFNγ frequency was 0.32% (range 0.07%–2.1%). For CD8+ T cells, median frequency of positive cells was 0.14% (range 0.01%–1.2%). Figure 2 shows representative flow analysis for three patients. Patient 1 had both a humoral and CMI response to past infection whereas patient 2 had only a humoral response, and patient 3 had no humoral or CMI response.

Overall, 11/22 (50%) and 8/22 (36.4%) patients had a positive pH1N1-specific CD4+ and CD8+ T cell response respectively. A CD4+ IFNγ response was present in 3/7 (42.9%) of seroprotected patients but also 7/12 (58.3%) patients who had negative serology (p = NS). Similarly, a CD8+ IFNγ response was present in 6/7 (85.7%) of seroprotected patients and 9/12 (75%) of patients with negative serology (p = NS). Four of 19 patients (21.1%) were negative for all three immunological parameters. The three patients with indeterminate serology (i.e. cross-reactive antibody to A/Brisbane) had a CMI response in 1 of 3 patients for CD4 and 2 of 3 patients for CD8. Of the three patients that were immunized for pH1N1, only one had a persisting CD4+IFNγ and CD8+IFNγ response at the time of follow-up.

**Figure 3:**
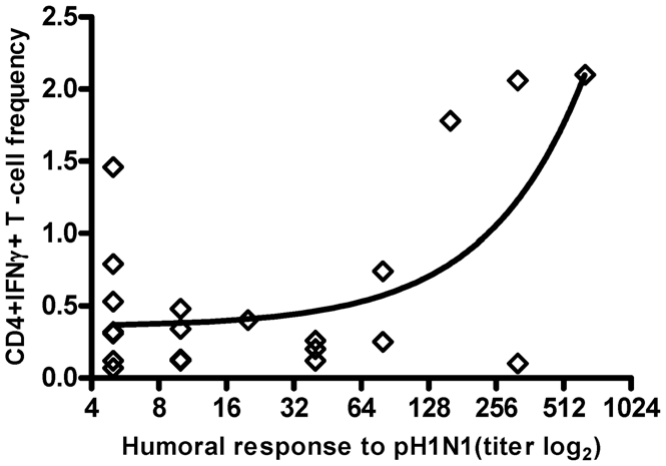
Relationship between CD4+IFNγ T-cell frequency (%) and the humoral response (n = 19). (R^2^ = 0.428, P = 0.002).

The CD4+ T cell response had a significant association with pH1N1 antibody titer; R^2^ = 0.428, p = 0.002 (Figure 3). Tacrolimus trough levels were inversely correlated with frequencies of CD4+IFNγ T-cells, i.e, CD4+IFNγ T-cell frequencies were higher in patients with lower tacrolimus trough levels (R^2^ = 0.442, p = 0.007). No other correlations with immunosuppression were observed. There was no significant correlation between CD4+IFNγ or CD8+IFNγ frequency of T cell specific cells and the presence of lymphopenia, time from transplant to infection or the use of early antiviral therapy. In addition, hospitalization at the time of natural infection was not predictive of a T-cell response.

## Discussion

We determined the long-term humoral and cellular immune responses in 22 adult transplant patients who recovered from pH1N1 infection. We found that only approximately one-third of our cohort had a sustained humoral response. Cellular responses were present in a similar proportion of patients (50% CD4 and 36.4% CD8) and a significant proportion of patients had an absence of both humoral and cellular (CD4, and CD8) responses (21.1%). Our results suggest that even after natural pH1N1 infection a substantial portion of transplant patients would be at risk for re-infection with the same strain in subsequent influenza seasons. To our knowledge, this is the first study examining the long-term responses to natural influenza infection in solid organ transplant recipients.

Another study looked at humoral and cellular immunity to pH1N1 in patients following hematopoietic stem cell transplantation. Only short-term immunological responses were evaluated (within 12 weeks of infection); the authors found that only 6/11 (54.5%) patients had a humoral response compared to 100% of controls and that H1N1-specific T cells could be measured in only 2/8 (25%) patients compared to 4/4 in the control group [13]. A single study has looked at the humoral response to infection in a cohort of immunocompetent patients infected with pH1N1. This study found a 100% seroconversion rate by day 30 in young adults but a rapid decline in titers to 52% by day 180 [4]. The decrease in titers over time is also shown in our study where the percentage of seroprotected patients was lower with increasing time post-infection. Our seropositivity rate at around the six month time point was quite similar to that shown by Wang et al.

Cellular immune responses to pandemic H1N1 infection have previously been described in immunocompetent patients by Bermejo-Martin *et al.* IL-8, IFNγ, IL-13, IL-10 were significantly higher in hospitalized patients vs. outpatients and controls. While both critical and non-critically hospitalized patients showed higher levels of IL-17 and TNFα than controls, only severe critically ill patients had significantly elevated levels of IL-17 and TNFα [8]. In the current study, we did not measure the cellular response at the time of infection but did find that hospitalized patients did not have a greater long-term cellular response. In transplant recipients, cellular responses have been investigated after vaccination but not after infection. For example, Mazzone et al. evaluated CMI in 43 lung transplant recipients and in 21 healthy controls after influenza vaccination. IL-2, IL-10, IFNγ, and granzyme B levels did not rise from pre- to post-vaccination in the lung transplant group and were overall lower in the transplant group when compared to the control group [14].

We also found that the ten heart and lung transplant recipients tended to have lower cellular responses to influenza infection compared with other types of transplant. This may reflect a higher net state of immunosuppression and is similar to that seen in influenza vaccine studies that suggest poor vaccine immunogenicity in lung transplant recipients compared to kidney recipients [15], [16]. In our study, higher tacrolimus levels were associated with a lower CD4+ T cell response although this was not the case for CD8 responses. We also found a significant association between CD4+ T cell response and the humoral response. In contrast, no correlation between cellular and humoral immunity has been found in studies of transplant patients who received seasonal influenza vaccine [17].

One limitation of our study is the small number of patients tested; to carry out similar studies in larger number of transplant patients with natural influenza infection, a multicenter approach would likely be needed. We did not do pre-infection testing or testing at earlier time points post-infection; however, similar to seroprevalence studies, we did serology for A/Brisbane to rule out cross-reactive H1N1 antibodies. We were also able to show diminished long-term response which is important when considering a revaccination strategy for subsequent annual vaccination. However, our findings represent an immune response to the infecting strain and not to significantly shifted influenza strains.

Although the decline in humoral immunity shown in our study is similar to that shown by Wang et al. in the immunocompetent population, we believe this is of significant concern in the immunocompromised transplant patient. From previous literature, transplant patients are more likely to have a poor outcome from influenza infection and more likely to have greater tissue viral loads and prolonged shedding times [1], [18]. Preventative strategies for re-infection include vaccination, antiviral prophylaxis, and safe-living strategies to avoid potential exposures. The immunogenicity of trivalent inactivated influenza vaccine after transplantation is quite variable and seroprotection post-vaccination ranges from 28–93% [9], [16]. In our study, the two patients that received pH1N1 vaccine after infection did not have a long-term response. Strategies such as giving two or three doses of influenza vaccine during the season have not been shown to significantly increase immunogenicity [16], [19]. Giving low-dose intradermal vaccine also did not improve responses in a cohort of lung transplant recipients [15]. Another strategy may be to provide chemoprophylaxis to all or a select group of transplant recipients during the influenza season. The cost-effectiveness, drug interactions, medication compliance and risk of antiviral resistance with this strategy would have to be considered. In summary, this study provides novel evidence that the majority of transplant patients with previous pH1N1 infection likely remain at risk for re-infection and are candidates for future prevention strategies.
